# Palliative Extubation in Pediatric Patients in the Intensive Care Unit and at Home: A Scoping Review

**DOI:** 10.1155/2023/6697347

**Published:** 2023-11-28

**Authors:** Joana Neto, Hugo Jorge Casimiro, Paulo Reis-Pina

**Affiliations:** ^1^Faculty of Medicine, University of Lisbon, Portugal; ^2^Hospital Palliative Care Team, Setúbal Hospital Centre, Setúbal, Portugal; ^3^Bento Menni's Palliative Care Unit, Casa de Saúde da Idanha, Sintra, Portugal

## Abstract

**Aim:**

This scoping review is aimed at systematically mapping the evidence on palliative extubation in the pediatric intensive care unit.

**Methods:**

MEDLINE, EBSCO, and Cochrane databases were searched for articles published between January 2018 and December 2022, in English. Critical appraisal of sources of evidence was done using the Joanna Briggs Institute tools. PRISMA guidelines for scoping reviews were followed.

**Results:**

Six studies were included, with 366 patients, from the USA (*n* = 4), Brazil (*n* = 1), and Germany (*n* = 1). Three were high-quality studies, two were moderate, and one was a low-quality study. Most studies were retrospective analysis; two were narrative approaches; two were evidence-based recommendation and quality improvement project; one study was a prospective intervention.

**Conclusion:**

Symptom control is crucial pre- and postextubation. A checklist (symptom management and family support) and a postdebriefing template improve team communication and staff support postextubation. Critical care transports from the hospital are feasible to provide extubation at home. A framework addressing common planning challenges and resource management is recommended for extubation at home. The provision of pediatric palliative extubation is necessary since futile measures and prolongation of suffering violate the principle of nonmaleficence. Future research on this subject will result in more benefits for patients, parents, and professionals.

## 1. Introduction

### 1.1. Rationale

In the last decades, the profile of hospitalised children has changed, with an increase in children with chronic diseases or technology-dependent conditions [1]. Although the survival of children with life-limiting conditions has improved [2], some of these children are admitted to the intensive care unit (ICU), and their end-of-life brings a lot of complexity in decision-making [3]. Children with limited life expectancy need individualised and comprehensive care [4].

In this scenario, limitation of treatment including withholding or withdrawing of life support measures, especially compassionate or palliative extubation (PALEXT), should be considered. PALEXT is a complex procedure defined as the interruption of mechanical ventilation in patients with irreversible and untreatable conditions, where the suffering associated with the futile prolongation of the death process outweighs the benefits for the patient [5].

The decision to whether withdraw life support is of great complexity, as it involves the patient, the family, and the medical staff, as well as ethical and moral questions. While discussing such a scenario, it is paramount to keep in mind the best interest of the patient, based upon the ethical principles of autonomy, justice, and proportionality [6].

Several studies have described PALEXT in adults [7–9]. However, there are few published studies involving children on this subject. Therefore, further evidence and review of evidence is still needed to assess the effects of such care, as well as its barriers.

### 1.2. Objectives

The aim of this scoping review was to systematically map the evidence on PALEXT in the pediatric population admitted to the ICU.

## 2. Methods

### 2.1. Protocol and Registration

This scoping review was not registered.

In this review, we followed the “Preferred Reporting Items for Systematic reviews and Meta-Analyses” statement, extension for scoping reviews [10], and we presented this checklist as a supplementary file.

### 2.2. Eligibility Criteria

We included studies according to these criteria:

- *Population*: patients of pediatric age, of any gender, with incurable or terminal diseases, according to the judgement of medical staff. We followed the policy statement by the American Academy of Pediatrics which considers 21 years as the upper limit of age [11]
- *Intervention*: studies that included PALEXT as the main intervention
- *Study design*: any. Considering the scarcity of literature on PALEXT in the pediatric population, we included as many studies as possible

The exclusion criteria comprised unspecified population (e.g., no age limits provided) or studies involving both pediatric and adult patients.

### 2.3. Information Sources

MEDLINE, EBSCO, and Cochrane databases were searched for articles published between January 1, 2018 and December 31, 2022. The sources were last searched for on January 3, 2023. No manual search was performed. The article authors were not contacted.

### 2.4. Search

The electronic search strategy included the following terms: Pediatric^∗^ AND (“palliative extubation” OR “terminal extubation” OR (“compassion^∗^” AND (“airway extubation” OR (“airway” AND “extubation”) OR “airway extubation” OR “extubat^∗^))). Filters were applied for articles in English, published in the last five years.

### 2.5. Selection of Sources of Evidence

**Figure 1:**
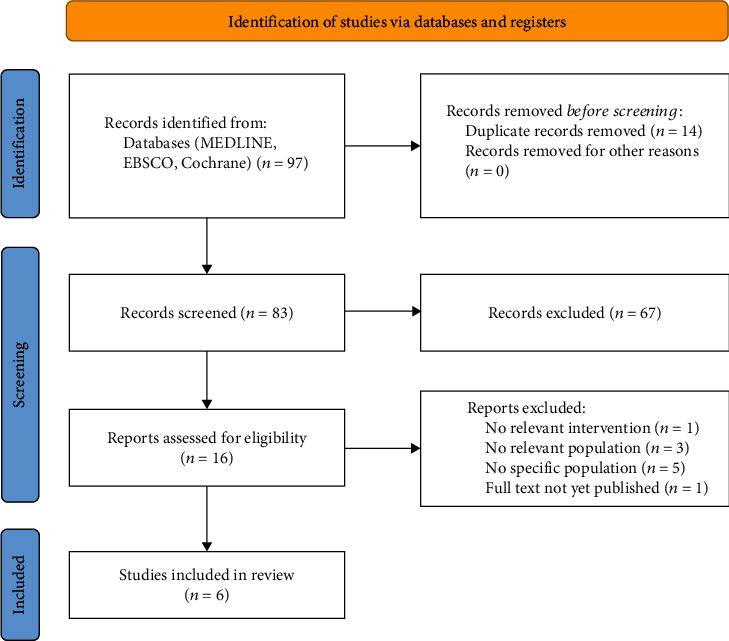
Flow diagram.

All the identified titles and abstracts were screened by the first author. The potentially eligible articles were selected for full-text analysis by two independent reviewers. Any disagreement between reviewers was resolved by discussion, until consensus was reached. No automation tools were used. The process of study selection is described in Figure 1 in a flow diagram [12].

### 2.6. Data Charting Process

A data-charting form was developed by two reviewers who decided which variables to extract. The two reviewers independently charted the data (in a Microsoft® 365 Excel Spreadsheet Software) and continuously updated the form in an iterative process. For the verification of the data and discussion of the results, a third person was involved.

### 2.7. Data Items

We abstracted data on article characteristics as follows: authors, year of publication, country (where the study took place; if patients were not included: the country where main authors worked in), study design, objectives, population, and main results.

During the writing of the narrative synthesis, the authors removed the data from the charting form and chose to present only the description of each article. The final version of the data-charting form is presented as a supplementary file (Appendix 1).

### 2.8. Critical Appraisal of Individual Sources of Evidence

Two independent reviewers participated in this process.

The Joanna Briggs Institute (JBI) Critical Appraisal tools were used for systematic and narrative reviews [13], cohort studies and comparative retrospective chart/registry studies [14], qualitative studies [15], and case series [16],

The mixed methods appraisal tool was applied to studies with diverse methodologies [17].

### 2.9. Synthesis of Results

We grouped the studies in three topics: (1) “Palliative Extubation in the Intensive Care Unit”, (2) “Palliative Extubation at home”, and (3) “Health care professionals' experiences and practices related to Palliative Extubation.”

Evidence was presented in a narrative format.

**Figure 2:**
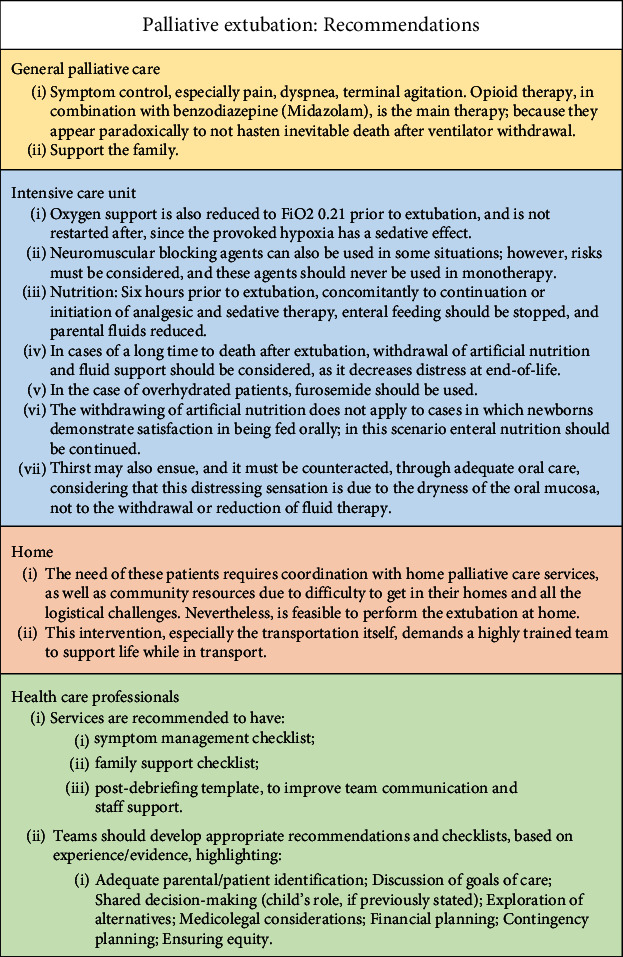
Recommendations for palliative extubation.

We summarized the evidence, namely, the most important recommendations on PALEXT originated from this scoping review in Figure 2.

## 3. Results

### 3.1. Selection of Sources of Evidence

Ninety-seven records were identified. Fourteen duplicates were removed. Eighty-three records were screened, and 67 were excluded after title/abstract analysis. The remaining 16 reports were assessed for eligibility, through a full-text analysis, and 10 reports were excluded. Six studies remained and were selected for this review.

The study selection is summarized in Figure 1.

### 3.2. Characteristics of Sources of Evidence

We included six studies from two continents: the USA (*n* = 4) [18–21], Brazil (*n* = 1) [22], and Germany (*n* = 1) [23]. Participants were 366 (348 patients retrospectively and 18 prospectively). Some studies used several methods in the same research. Most of the studies presented retrospective analysis [18–21], two had a narrative approach [20, 23], and two presented an evidence-based recommendation (framework and checklist) [20], or quality improvement project (checklist and debriefing sheet) [21]. Only one study has a 6-month prospective intervention analysing 18 cases of PALEXT, with the participation of nurses and other healthcare professionals [21].

The study of Garcia et al. (USA, 2021) is a case series presenting three critical care transports from the Pediatric Cardiac ICU to provide PALEXT at home [18].

The study of Winter et al. (USA, 2021) is a retrospective cohort study with 237 patients who died after PALEXT, and the objectives were (1) to train a memory model to predict cardiac death 1-hour within PALEXT, (2) to calculate the positive predictive value of the model and the “number needed to alert” for potential organ donors, and (3) to examine associations between time to cardiac death and patients' characteristics and physiologic variables [19].

The study of Woodruff et al. (USA, 2021) is a narrative review aimed at developing an experience-based framework and checklist for PALEXT at home for critically ill children at the end-of-life, addressing common planning challenges and resource management [20].

The study of Donoho et al. (USA, 2021) is a retrospective review and a quality improvement project directed at nursing staff and is aimed at developing and implementing a symptom management and family support checklist and postdebriefing template to improve team communication and staff support at a Neonatal ICU [21].

The study of Affonseca et al. (Brazil, 2020) is a retrospective analysis conducted in a Brazilian pediatric hospital, reporting a five-year experience of 19 patients (aged five months to nine years) permanently dependent on ventilatory support submitted to PALEXT [22].

The study of Garten and Bührer (Germany, 2019) is a narrative review of nonpharmacological/pharmacological measures for pain and distress management in palliative neonatal care. It includes some recommendations on PALEXT [23].

More characteristics of sources of evidence are presented in Appendix 1 (Supplemental File).

### 3.3. Critical Appraisal within Sources of Evidence

This scoping review included three high-quality studies [18, 21, 22], two moderate-quality studies [19, 20], and one low-quality study [23]. We chose to include the latter study because when the critical appraisal tool was applied, there was only one “no” item and seven “unclear” items. All authors considered that this study should be included, moreover due to the scarcity of research on PALEXT.

The critical appraisal of the six studies is summarized in Table 1.

### 3.4. Results of Individual Sources of Evidence

Garcia et al. described a series of three cases [18]. The first patient is a 7-month-old male who remained hemodynamically stable during transport (distance = 80 miles), and the hospice team assumed care. His code status was kept “do not resuscitate.” He survived for several years, but the exact survival time is absent. The second patient is a 9-month-old infant who remained stable during transport (a 5-mile 15-minute trip) and died 11 minutes after extubation; he was under sedation pre- and post-PALEXT. The third patient is a 19-year-old female whose transport was a 55-mile, 120-minute trip. She died two hours after extubation and developed some agonal breathing that required extra sedation [18].

In Winter et al.'s study, the population (237 patients aged 0-21 years) died at the Pediatric or Cardiothoracic ICU after PALEXT [19]. The long short-term memory model developed had a positive predictive value of 0.81 and a sensitivity of 94% in predicting death within 1-hour of PALEXT. Significant predictors of 1-hour death included low Glasgow Coma Scale, high PaO2 to FiO2 ratio, low pulse oximetry, and low serum bicarbonate. The model identified 93% of potential organ donors with a number needed to alert of 1.08. Approximately 39% of patients met organ procurement and transplantation network criteria for liver and kidney donors [19].

Woodruff et al. presented a summary of literature including [20] (1) ten case reports/series involving sixty patients, all reporting the feasibility of PALEXT in the ICU; (2) three evidence-based recommendations presenting checklist, palliative transport, care pathway, and recommendations on PALEXT; (3) one policy statement (American Academy of Pediatrics) which covers informed consent, pediatric assent, and autonomy in children and adolescents; (4) four studies (three surveys and one expert recommendations) about palliative goal setting; and (5) one review addressing children's autonomous decision-making and assent/consent in pediatric ICU [20]. The authors presented an evidence and experience-based recommendation for PALEXT at home, based upon a framework and associated checklist. They highlighted the patient identification and goal exploration, exploration of alternatives, child's role in shared decision-making, financial planning, medicolegal considerations, contingency planning, and ensuring equity.

Donoho et al. included (1) patients who underwent PALEXT in Neonatal ICU and (2) healthcare professionals who cared for patients who underwent PALEXT [21]. Out of 50 total deaths during the initial 12-month review, 29 (58%) occurred following PALEXT. Additionally, during the 6-month intervention period, 18 PALEXT events occurred. Common indications for redirection of care toward PALEXT in the baseline group were severe neurologic impairment and multiorgan failure (both 31%) and in the postintervention group were cardiopulmonary failure (100%). Regarding the quality improvement project, 43 nurses who cared for PALEXT patients participated in the baseline assessment survey, and 11 in the postintervention survey. In postevent surveys, 66 staff people participated, 82% reported using the checklist during PALEXT, and 68% reported reviewing the checklist with the medical team. The ability to locate the PALEXT checklist varied by role: more attendings and fellows than nurses of respiratory therapists. The checklist and debriefing were helpful for ensuring staff support during a PALEXT event [21].

Affonseca et al. in a series of 19 patients showed that most of the extubations (68.4%) were performed in the ICU, and 11 patients (57.9%) died in the hospital [22]. Thirteen patients used an orotracheal tube, and the others used tracheostomy. Main symptoms were dyspnea and pain, and the main drugs were opioids and benzodiazepines. Eight patients did not receive medication pre-PALEXT; nine patients received corticosteroids, six atropine, and five scopolamine. No discomfort symptoms were observed in eight patients post-PALEXT, but five people received morphine and one patient, fentanyl. The duration of mechanical ventilation, use of endotracheal tube or tracheostomy cannula, the offer of respiratory support after extubation (oxygen or noninvasive ventilation), and the ventilatory parameters before extubation were not associated with in-hospital death [22].

Garten and Bührer affirmed that regarding pain and distress, prevention is the most important fact [23]. Behavioural (e.g., swaddling and facilitated tucking) and physical (e.g., kangaroo care and breastfeeding) strategies significantly reduce physiological and behavioural pain reactions of newborns. Sucrose also plays a role in analgesia. No analgesics with proven efficacy and safety other than opioids are currently available for the systemic treatment of severe pain in neonates. The dose of opioids should be titrated individually to the effect. There is insufficient evidence on analgesic efficacy or safety for acetaminophen, nonsteroidal anti-inflammatory drugs, ketamine, or transdermally administered local anaesthetics in neonates [23]. Regarding acute dyspnea, starting opioid therapy is adequate to prevent pain and distress before PALEXT. Benzodiazepines may be used as anxiolytics or adjuvant to opioid therapy. Opioids and benzodiazepines appear paradoxically to not hasten inevitable death after ventilator withdrawal. Regarding sedation, conscious sedation is often ideal, but in case of intense distress, severely refractory symptoms, or anticipated death within hours, continuous deep sedation is indicated [23]. In the absence of controlled trials, the choice of sedatives is largely empirical (benzodiazepines, barbiturates, and alpha2-adrenoreceptor agonists). Combining opioids with benzodiazepines (e.g., midazolam) is recommended. Phenobarbital may be an alternative, given preferably orally at 5 mg/kg/dia. Neuromuscular blocking agents should never be used as a sole agent, and deep analgo-sedation should be warranted [23].

### 3.5. Synthesis of Results

#### 3.5.1. Studies on “Palliative Extubation in Intensive Care Units”

*(1) Symptom Control*. The management of neonates who are candidates to PALEXT in the ICU obliges to an anticipation of pain and terminal agitation, as well as prophylactic or rapid treatment of dyspnea. Analgesia and sedation should be the top priorities in controlling symptoms, especially dyspnea and pain. Opioid therapy, sometimes in combination with benzodiazepine, is the main therapy used in symptom control [23].

Six hours prior to PALEXT, concomitantly to continuation/initiation of analgesic and sedative therapy, enteral feeding should be stopped, and parental fluids reduced, and in the case of overhydrated patients, furosemide should be used. Oxygen support should be reduced to FiO2 0.21 pre-PALEXT and not restarted afterwards, since the provoked hypoxia has a sedative effect [23].

In cases of a long time to death post-PALEXT, withdrawal of artificial nutrition and fluid support should be considered, as it decreases distress at end-of-life. This consideration does not apply to cases in which newborns demonstrate satisfaction in being fed orally. Thirst may also ensue, and it must be counteracted, through adequate oral care, considering that this distressing sensation is due to the dryness of the oral mucosa, not to the withdrawal or reduction of fluid therapy [23].

*(2) Time to Death Postextubation*. This topic varied throughout studies.

Winter et al. in a cohort study (*n* = 237) found that the time to death after PALEXT was 0.3 hours (interquartile range, 0.16–1.6 hours). Approximately 70% of the patients died within one hour, and the remaining 30% had a median time to death of 5.8 hours [19].

Donoho et al. in a retrospective cohort of 29 cases found a median time PALEXT to death of 01 h 02 min (ranging from one minute to 33 days and 14 hours). During the 6-month intervention period, the 18 neonatal PALEXT cases had the median time to death after PALEXT that was 01 h 22 min (ranging from 18 minutes to two days, 21 hours and 18 minutes) [21].

Affonseca et al. in a retrospective analysis of 19 cases of PALEXT showed eleven in-hospital deaths and eight out-hospital deaths. The time between PALEXT and in-hospital death was 15 minutes to five days (median = 04 h 20 min). No identification of predictors associated with in-hospital death was found. For the patients who were discharged and died, three died between 50 and 214 days post-PALEXT in the first year after discharge, and one patient died two years after discharge (no mention to the time between PALEXT and death). There was no available data regarding the other four patients [22].

#### 3.5.2. Studies on “Palliative Extubation at Home”

One study focused on a case series of three palliative critical home transports from the ICU [18]. This intervention, especially the transportation itself, demanded a highly trained team to support life while in transport. The need of these patients required coordination with home palliative care services, as well as community resources due to difficulty to get in their homes and all the logistical challenges. Once at home, PALEXT was performed, one patient died 11 minutes afterwards, another two hours later and one remained alive for several years [18].

A framework on PALEXT at home was presented as an option for end-of-life care [20]. It consisted of preparatory steps (planning responsibilities), a checklist for PALEXT with shared roles, and follow-through steps (activities which encourage support of the team and family and incorporate feedback). The checklist should begin as early as possible in advance of planned transport. Any out-of-pocket costs that could not be eliminated should be discussed with the family. Overall, PALEXT at home is feasible for pediatric ICU patients but could be difficult to orchestrate, and it could relieve suffering and improve the quality of the dying experience for a child [20].

#### 3.5.3. Studies on “Attitude of Health Personnel about Palliative Extubation”

Donoho et al. developed and implemented a novel symptom management and family support checklist and postdebriefing template to improve team communication and staff support [21]. Eighteen events were studied. There were 54 respondents who located and used the checklist, whose roles included attending neonatologists or hospitalists, nurses, neonatology fellows, respiratory care practitioners, and others. They assessed the checklist and debriefing as helpful because (1) a provider from the medical team was available and near bedside during and in the short-term after PALEXT to help ensure adequate symptom management (96%), (2) it ensured staff support during PALEXT (80%), (3) the medical team was effective in providing anticipatory guidance to families prior to PALEXT (78%), (4) it ensured family support during PALEXT (74%), (5) it ensured team communication after PALEXT (56%), and (6) it ensured staff support after PALEXT (56%). The responding nurse staff at the neonatal ICU, endorsing “good” communication with the medical team, increased by 60%, and the debrief participation rate improved by 96%; however, similar responses were reported at baseline and postintervention for questions assessing perceived levels of patient comfort. Over time, the percentage of survey responders who agreed that these tools were helpful almost doubled [21].

### 3.6. Summary of the Main Recommendations

A synthesis of the main recommendations on PALEXT from a palliative care point of view is presented in Figure 2.

## 4. Discussion

### 4.1. Summary of Evidence

The evidence on PALEXT is scarce, specifically in the pediatric population.

In this scoping review, we found no standardization or guidelines on the planning and management of this extremely specialized form of care in the ICU. One study presented the results of a quality improvement initiative and a novel symptom management and family support checklist and postdebriefing template to improve team communication and staff support [21]. Unfortunately, neither the checklist nor the postdebriefing template was presented in the article. This initiative improved team communication and support since professionals might feel educationally and emotionally unprepared for this high-impact event.

Another article defended the importance of palliative care on preventing and relieving suffering by reducing the severity of disease symptoms [23]. Furthermore, recommendations were given focusing on PALEXT in neonatal care settings. The International Standards for Pediatric Palliative Care affirms that (1) hospitals providing neonatal and maternal care need to develop perinatal palliative care pathways, and (2) palliative care may be provided in the delivery room, postpartum ward, in the neonatal ICU, at home, or wherever is thought to be most appropriate and provided that this approach is consistent with family goals of care [24]. Families might believe that they are responsible for their relative's death; therefore, it is crucial to state that what causes the death is the disease itself, not the decision to perform PALEXT, absolving them from this responsibility [25]. In the end-of-life, new goals of care should be discussed, such as maintaining the child's quality of life, or deciding the location of the child's end-of-life care and death [26]. Occurrence of death outside of the hospital environment could represent a viable alternative for terminally and critically ill, technology-dependent ICU patients [18]. Sadly, it is likely that the high proportion of hospital deaths currently reported reflects not that choice is unavailable, but that choice is not offered [27].

The standard environment at the end-of-life where PALEXT usually takes place is the hospital, more often the ICU; however, recent emphasis has been given to alternative environments, such as the child's home, and this option should be considered and provided to the families in line with a family-centred care [18, 20]. Relatives might prefer it, since it is a familiar environment provided with a greater sense of privacy, comfort, security, control, and closure for the family, which in turn might help with bereavement [18, 22, 28, 29].

If home is the family's chosen place of death, pediatric intensivists should provide PALEXT not in the ICU, but at home, as an option for end-of-life care [18, 20]. In this case, preparation begin as early as possible in advance of planned transport [20]. Pediatric palliative critical care transports from the ICU to home or hospice are an uncommon occurrence and perhaps an underutilized option [18]. This scenario brings an additional level of complexity, well reflected on the transportation and home care process, with a new and vast plethora of variables to be considered, such as unpredictability [22, 30, 31]. Garcia et al. described three palliative critical care transports from the cardiac ICU for PALEXT at home as a compassionate alternative for families with terminal and critically ill children [18]. These transports are feasible and a valuable option available for their families seeking optimal out-of-hospital end-of-life care for their critically ill and technology-dependent child [18]. Parents' decision to transport the child home from the ICU is a process motivated by promises to the child or a conviction that it was the right thing to do [28]. Being home provided time with the child in the context of their family and contributed to their lives. Memories of the experience brought comfort and a sense of fulfillment. The parents were gratified by the attention to safety and detail involved, but the actual transport home was stressful to them [28].

Knowing that PALEXT at home was feasible for pediatric ICU patients but could be difficult to orchestrate, Woodruff et al. presented an experience-based recommendation for PALEXT at home [20]. This was the only article that really showed the framework and associated checklist. It consisted of preparatory steps (including critical planning responsibilities), a checklist for PALEXT with shared roles, and follow-through steps (including activities which encourage support of the team and family and incorporate feedback into continuous quality improvement activities). They highlighted the patient identification and goal exploration, exploration of alternatives, child's role in shared decision-making, financial planning, medicolegal considerations, contingency planning, and ensuring equity [20].

PALEXT constitutes an extremely specialized form of care and demands the support of a well-prepared and trained multidisciplinary team. PALEXT can be a stressful experience for both families and staff in the ICU. Any time staff participate in the withdrawal of life-sustaining medical treatment, there may be moral distress [21]. Support for staff by the palliative, ethics, spiritual care, and bereavement teams can be helpful. Opportunities for debriefs should be made accessible to all staff [20]. In this scoping review, we found no studies about the perceptions, fears, and myths of staff on PALEXT. There is some literature on staff comfort with end-of-life care in the ICU, mainly represented by the nursing population [32], though other staff members, such as physicians, respiratory therapists, and social workers, are presumed to face many of the same challenges. This topic merits more research in the near future.

The evidence that exists is very heterogenous and is mostly qualitative which limited the analysis. The only quantitative variable that was comparable, across three studies, was the time to death after PALEXT [19, 21, 22]. This great limitation proves and implicates the need for future work in this area, specially a more rigorous one, which paradoxically can be interpreted as a strength by calling for attention on the topic.

### 4.2. Limitations

We found a scarce number of studies on PALEXT in neonatal ICU settings.

Most articles were American, with little representation from Europe and none from other continents.

Most studies had small samples, and the process of PALEXT was not fully described.

Considering the ample diversity of study designs, objectives, and outcomes, the results are greatly variable and heterogenous and cannot be extrapolated.

Considering the scarcity of the topic, broader eligibility criteria were sounder. We excluded research with no complete paper publication or unpublished (gray) literature, and we did not hand-search key journals nor did scan reference sections. This is another limitation of our study.

## 5. Conclusion

The provision of PALEXT in selected pediatric patients is of the utmost importance, and in an era dominated by technological improvements, the reality of futile measures and prolonging of suffering should not be overlooked. Discouragingly, no standardization or guidelines was found on the planning and management of PALEXT in the ICU, but a framework and checklist were proposed for PALEXT at home.

More research is necessary on PALEXT and palliative care in the ICU, namely, indications, protocol, checklist, neonatal end-of-life anticipatory guidance, symptom management, legal considerations, place of dying and death, clinical monitorization pre- and postintervention, staff and family support, healthcare professional education on end-of-life, effective communication (between staff members and between staff and family members), grief preparation, strategies to combat compassion fatigue, and debriefs pre- and postintervention, amongst others.

We consider future research on PALEXT to be extremely important, primarily due to the paucity of evidence and secondarily to enable guidelines and consensus which can only be derived from a better understanding of the subject. The result can be a more specialized, legitimate and skillful provision of palliative care, even in highly technological environments such as the ICU.

## Figures and Tables

**Table 1:** Summary of the critical appraisal of the individual sources of evidence (*n* = 6).

Study	The Joanna Briggs Institute Critical Appraisal tools						
	Total number of items	Number of “YES” items	Number of “NO” items	Number of “UNCLEAR” items	Number of “NOT APPLICABLE” items	Risk of bias	Degree of quality
Garten et al., [23]	11	3	1	7	0	High	Low
Affonseca et al., [22]	11	11	0	0	0	Low	High
Garcia et al., [18]	11	8	0	1	1	Low	High
Winter et al., [19]	11	5	2	0	4	Moderate	Moderate
Woodruff et al., [20]	10	4	3	2	1	Moderate	Moderate

	The mixed methods appraisal tool						
	Total number of items	Number of “YES” items	Number of “NO” items	Number of “Cannot TELL” items		Risk of bias	Degree of quality
Donoho et al., [21]	12	12	0	0		Low	High

## Additional Points

*Key Notes*. (i) In pediatric palliative care, symptom control is crucial pre- and postextubation, and critical care transports from the hospital are feasible to provide extubation at home. (ii) A checklist (symptom management and family support) and a postdebriefing template improve team communication and critical care staff support postextubation. (iii) A framework and checklist, addressing common planning challenges and resource management, are recommended for extubation at home for critically ill children at the end-of-life.

## Supplementary Materials

Supplementary 1 The characteristics of each source of evidence are provided with more details in a table (Appendix 1). Likewise, all relevant outcome data for each source of evidence are also included in Appendix 1.

Supplementary 2 The checklist of the PRISMA extension for scoping reviews is also presented as a supplementary file.
