# Supplementary material for: Palliative Extubation in Pediatric Patients in the Intensive Care Unit and at Home: A Scoping Review
Source: Int J Pediatr. 2023 Nov 28;2023:6697347. doi: 10.1155/2023/6697347 (PMC10697771; doi:10.1155/2023/6697347)
Supplement: Supplementary 1 — The characteristics of each source of evidence are provided with more details in a table (Appendix 1). Likewise, all relevant outcome data for each source of evidence are also included in Appendix 1. [file 6697347.f1.pdf]

Appendix 1 - Characteristics of the individual studies (n=6)

Authors and Year of publication	Country of origin	Type of study	Objectives	Population
**Garten et al., (2019)**	Germany	Narrative Review	Review non-pharmacological and pharmacological measures for pain and distress management in the context of neonatal ICU focusing on PALEXT.	No participants
	**Main Results**			
	- Prevention of pain and distress is the most effective way of successful pain and distress management.<br><br>- No neonatal pain scale has been explicitly validated for use in patients under palliative neonatal care.<br><br>- The combination of several non-pharmacological measures is superior to the isolated use of nonpharmacological measures, for reducing neonates' stress reactions in acute procedural pain.<br><br>- Sucrose can be used for analgesia, particularly for acute procedural pain relief.<br><br>- No analgesics with proven efficacy and safety other than opioids are currently available for the systemic treatment of severe pain in neonates, however they are not very effective in producing reliable, prolonged sedation in severely distressed infants under palliative care. Combining opioids with specific sedatives such as benzodiazepines (Midazolam) is recommended.			

	- Opioid therapy to prevent pain and distress caused by acute dyspnea should be started before withdrawal of mechanical ventilation and compassionate extubation. Benzodiazepines may be used as anxiolytics or as an adjuvant to an opioid analgesic. Opioids and benzodiazepines appear paradoxically to not hasten inevitable death after ventilator withdrawal.<br><br>- No sufficient study data are available for the neonatal age for prophylaxis and treatment of side effects under opioid therapy.<br><br>- It is not possible to provide general or weight-related standard and/or maximum dosages, regardless of the type of application used.<br><br>- The targeted level of sedation should be the lowest that relieves distress. Conscious sedation is often considered the ideal level of sedation. In case of intense distress, severely refractory symptoms, or anticipated death within hours, continuous deep sedation may be indicated.<br><br>- There is insufficient scientific evidence on analgesic efficacy or safety for the use of acetaminophen, non-steroidal anti-inflammatory drugs, ketamine or transdermally administered local anaesthetics in neonates.<br><br>- Medications widely used to deliver sedation in palliative neonatal care are opioids, benzodiazepines, barbiturates, and alpha2-adrenoreceptor agonists.<br><br>- Phenobarbital may be used alternatively to midazolam, given preferably orally 5mg/kg/day.<br><br>- Very little data is available on the use of alpha2-adrenoreceptor agonists in neonates. Studies available so far prove a sedative effect also in newborns.<br><br>- Should never be used as a sole agent and deep analgo-sedation should be warranted.<br><br>- Six hours before compassionate extubation, enteral feeding should be stopped and parenteral fluids reduced, overhydrated patients should be dehydrated with furosemide. At the same time administration of sedatives (for distress) and opioids (for pain and/or dyspnea) should be continued or started.

<table>
<tr><td></td><td colspan="4">- Oxygen supplementation goal is at FiO2 0.21 before terminating mechanical ventilation and not used after compassionate extubation.

Continuing artificial nutrition and hydration may not always be the indicated, treating thirst through adequate oral care is always necessary.</td></tr>
<tr><th>Authors and Year of publication</th><th>Country of origin</th><th>Type of study</th><th>Objectives</th><th>Population</th></tr>
<tr><td rowspan="3">Affonseca et al., (2020)</td><td>Brazil</td><td>Retrospective descriptive analysis of a series of patients submitted to palliative extubation</td><td>Present the characteristics of paediatric patients with chronic and irreversible diseases submitted to PALEXT</td><td>19 patients, aged 5 months to 9 years (mean age of 2.2 years), who were submitted to PALEXT between April 2014 and May 2019, at a public pediatric ICU in Brazil</td></tr>
<tr><td colspan="4">Main Results</td></tr>
<tr><td colspan="4">- 68.4% of extubation were performed in the ICU; 11 patients (57.9%) died in the hospital. The time between mechanical ventilation withdrawal and in-hospital death ranged from 15 minutes to five days with a median of four hours and 20 min.

- In the first year after discharge, three patients died between 50 and 214 days after extubation and one patient died two years after hospital discharge.

- The main symptoms were dyspnea and pain, and the main drugs used to control symptoms were opioids and benzodiazepines.

- The mean time during which patients received ventilatory support before extubation was 188 days among patients discharged from the hospital and 276 days among those who died during hospitalization.</td></tr>
</table>

	- Eight patients did not receive medication as preparation for extubation; nine patients received corticosteroids, six received atropine, and five received scopolamine. No discomfort symptoms were observed in eight patients after the palliative extubation, but five of them were receiving morphine and one patient, fentanyl.<br><br>- The duration of mechanical ventilation, use of endotracheal tube or tracheostomy cannula, the offer of respiratory support after extubating (oxygen or non-invasive ventilation), and the ventilatory parameters before extubation were not associated with in-hospital death.

**Authors and Year of publication**	**Country of origin**	**Type of study**	**Objectives**	**Population**
**Garcia et al., (2021)**	United States	Case series	Present palliative critical care home transports from the Paediatric Cardiac ICU	3 patients, 7-months-old male, 9 months-old infant, 19 years-old female
	**Main Results**			
	- 7 months patient: history of prematurity, hypoplastic left heart syndrome, multicystic dysplastic kidney, partial DiGeorge syndrome, and vocal cord paralysis. He later developed stenosis and thrombosis of the pulmonary arteries and right superior vena cava. Transport distance was 80 miles. During transport, the patient remained stable. The hospice team assumed care. Survived for several years.<br><br>- 9 months patient: trisomy 21 and complete atrioventricular canal defect with posterior severe biventricular diastolic dysfunction, diuretic resistance, and atrial fibrillation, hypothyroidism, acute kidney injury, adrenal insufficiency, and deep venous thrombosis of femoral and jugular veins. Transport distance was 5 miles, 15-minute trip. During transport, the patient remained stable. Died 11 minutes after extubation. Patient was under sedation prior and throughout withdrawal of support.			

	- 19 years old patient: history of Cockayne syndrome, with right heart failure, secondary to pulmonary hypertension complicated by bilateral pleural effusions and ascites. Transport distance was 55 miles, 120 minutes. Died 2 hours after extubation. Developed some agonal breathing, requiring extra sedation.			
**Authors and Year of publication**	**Country of origin**	**Type of study**	**Objectives**	**Population**
**Winter et al., (2021)**	United states	Retrospective cohort study	1) train a long short-term memory model to predict cardiac death within 1 hour after terminal extubation, 2) calculate "Positive predictive value" of the model and the number needed to alert among potential organ donors, 3) examine associations between time to cardiac death and the patient's characteristics and physiologic variables.	237 patients, aged 0–21 years old (mean age of 1.3 years) who were admitted to the Children's Hospital Los Angeles Pediatric ICU or Cardiothoracic ICU from January 2011 to December 2018 and died in the ICU after terminal withdrawal of invasive mechanical ventilation
	**Main Results**			
	- The median time to death after PALEXT was 0.3 hours; 70% of patients died within 1 hour.<br><br>- The long short-term memory model had a positive predictive value of 0.81 at a sensitivity of 94% when predicting death within 1 hour of PALEXT.<br><br>- About 39% of patients who died within 1 hour met organ procurement and transplantation network criteria for liver and kidney donors.<br><br>- The long short-term memory identified 93% of potential organ donors with a number needed to alert of 1.08.			

	- A Cox proportional hazard model identified independent predictors of shorter time to death including low Glasgow Coma Score, high Pao2-to-Fio2 ratio, low-pulse oximetry, and low serum bicarbonate.			
**Authors and Year of publication**	**Country of origin**	**Type of study**	**Objectives**	**Population**
**Woodruff et al., (2021)**	United States	Mixed: Narrative review and Evidence-based recommendation (framework and checklist)	Develop an evidence- and experience-based framework and checklist for PALEXT at home for critically ill children at the end of life, addressing common planning challenges and resource management.	Directed at terminally ill paediatric patients whose goal is at home PALEXT
	**Main Results**			
	The authors presented a Summary of Literature including:<br><br>1) ten case reports/series (nine studies from the USA and one from the UK; total 60 patients admitted to pediatric ICU; all reporting the feasibility of PALEXT).<br><br>2) three evidence-based recommendations (two studies from the USA and one from the UK, presenting checklist, palliative transport, care pathway, and recommendations on PALEXT).<br><br>3) one Policy statement from the American Academy of Pediatrics (Committee on Bioethics) which covers ethical basis for informed consent and pediatric assent including discussion of the role of developing autonomy in children and adolescents (not specific to end-of-life care).<br><br>4) four studies (three surveys and one expert recommendations) from the USA and the UK, about palliative goal setting.			

	5) one review addressing the changing views on children's autonomous decision-making in the context of assent/consent specific to care of children in the pediatric ICU.<br><br>The authors present a framework that is a conceptualization for pediatric intensivists providing PALEXT at home, as an option for end-of-life care. There are preparatory steps (including critical planning responsibilities), a checklist for PALEXT with shared roles, and follow-through steps (including activities which encourage support of the team and family and incorporate feedback into continuous quality improvement activities).<br><br>- Authors recommended that the checklist should begin as early as possible in advance of planned transport. At each step, one should assess potential out-of-pocket expense that may be incurred by the family and work to curtail or eliminate these costs with the help of case management. Any out-of-pocket costs that cannot be eliminated must be discussed with the family.<br><br>- PALEXT at home can relieve suffering and improve the quality of the dying experience for a child.<br><br>- PALEXT at home is feasible for pediatric ICU patients but can be difficult to orchestrate.<br><br>- The proposed framework and associated checklist for PALEXT at home may be useful but requires validation and adaptation for different cases and settings.			
**Authors and Year of publication**	**Country of origin**	**Type of study**	**Objectives**	**Population**
**Donoho et al., (2021)**	United states	Mixed Retrospective chart reviews and Quality improvement project (checklist and debriefing sheet)	Develop and implement a novel symptom management and family support checklist and post-debriefing template to improve team communication and staff support	Patients undergoing PALEXT in a North American Neonatal ICU between October 2017 and September 2018 + Health care personnel who cared for patients who underwent PALEXT at the Neonatal ICU

	Main Results
	- 50 total deaths in the Neonatal ICU during the initial 12-month chart review, 58% (n=29) occurred following PALEXT.<br><br>- During the 6-month intervention period, 18 PALEXT events took place.<br><br>- The most common indications for re-direction of care toward PALEXT in the baseline group were severe neurologic impairment and multiorgan failure (both 31%, n = 9); cardiopulmonary failure was the primary indication for every PALEXT event in the post-intervention group (100%, n = 9).<br><br>- Similar responses were reported at baseline and post-intervention for questions assessing perceived levels of patient comfort.<br><br>- Responses endorsing “good” communication with the medical team improved by 60%,<br><br>- Reported rate of participation in a post-event debrief improved by 96% improvement over time in percentage of survey respondents who agreed that the checklist and debriefing was helpful.

Legend: ICU – Intensive Care Unit; PALEXT – Palliative Extubation; UK – United Kingdom; USA – United States of America.
